# Increased Expression Profile and Functionality of TLR6 in Peripheral Blood Mononuclear Cells and Hepatocytes of Morbidly Obese Patients with Non-Alcoholic Fatty Liver Disease

**DOI:** 10.3390/ijms17111878

**Published:** 2016-11-10

**Authors:** María Teresa Arias-Loste, Paula Iruzubieta, Ángela Puente, David Ramos, Carolina Santa Cruz, Ángel Estébanez, Susana Llerena, Carmen Alonso-Martín, David San Segundo, Lorena Álvarez, Antonio López Useros, Emilio Fábrega, Marcos López-Hoyos, Javier Crespo

**Affiliations:** 1 Gastroenterology and Hepatology Department, Marqués de Valdecilla University Hospital, 39008 Santander, Spain; piruzubieta@gmail.com (P.I.); anpuente@humv.es (Á.P.); susanallerena@hotmail.com (S.L.); maika_1387@hotmail.com (C.A.-M.); digfge@humv.es (E.F.); javiercrespo1991@gmail.com (J.C.); 2 Infection, Immunity and Digestive Pathology Group, Research Institute Marqués de Valdecilla (IDIVAL), 390008 Santander, Spain; david.ramos.m@gmail.com (D.R.); aesga@hotmail.com (Á.E.); 3 Transplant and Autoimmunity Group, Research Institute Marqués de Valdecilla (IDIVAL), 39008 Santander, Spain; loraksc@gmail.com (C.S.C.); david_san_segundo@hotmail.com (D.S.S.); lorenalvar@hotmail.com (L.Á.); mlopezhoyos@humv.es (M.L.-H.); 4 Immunology Department, Marqués de Valdecilla University Hospital, 39008 Santander, Spain; 5 General Surgery Department, Marqués de Valdecilla University Hospital, 39008 Santander, Spain; alopez@humv.es

**Keywords:** TLR6, TLR2, non-alcoholic fatty liver disease, lobular inflammation, morbid obesity, pro-inflammatory cytokines

## Abstract

Current evidence suggests that gut dysbiosis drives obesity and non-alcoholic fatty liver disease (NAFLD) pathogenesis. Toll-like receptor 2 (TLR2) and TLR6 specifically recognize components of Gram-positive bacteria. Despite the potential implications of TLR2 in NAFLD pathogenesis, the role of TLR6 has not been addressed. Our aim is to study a potential role of TLR6 in obesity-related NAFLD. Forty morbidly obese patients undergoing bariatric surgery were prospectively studied. Cell surface expression of TLR2 and TLR6 was assessed on peripheral blood mononuclear cells (PBMCs) by flow cytometry. Freshly isolated monocytes were cultured with specific TLR2/TLR6 agonists and intracellular production of cytokines was determined by flow-cytometry. In liver biopsies, the expression of TLR2 and TLR6 was analyzed by immunohistochemistry and cytokine gene expression using RT-qPCR. TLR6 expression in PBMCs from non-alcoholic steatohepatitis (NASH) patients was significantly higher when compared to those from simple steatosis. The production of pro-inflammatory cytokines in response to TLR2/TLR6 stimulation was also significantly higher in patients with lobular inflammation. Hepatocyte expression of TLR6 but not that of TLR2 was increased in NAFLD patients compared to normal liver histology. Deregulated expression and activity of peripheral TLR6 in morbidly obese patients can mirror the liver inflammatory events that are well known drivers of obesity-related NASH pathogenesis. Moreover, TLR6 is also significantly overexpressed in the hepatocytes of NAFLD patients compared to their normal counterparts. Thus, deregulated TLR6 expression may potentiate TLR2-mediated liver inflammation in NAFLD pathogenesis, and also serve as a potential peripheral biomarker of obesity-related NASH.

## 1. Introduction

Non-alcoholic fatty liver disease (NAFLD) is characterized by an ectopic accumulation of fat in the liver, which is tightly associated with obesity and metabolic syndrome [1]. The spectrum of liver damage in NAFLD is wide, ranging from simple steatosis, with a predominant benign course, to non-alcoholic steatohepatitis (NASH), whose distinctive feature is the presence of lobular inflammation and hepatocellular ballooning that may eventually lead to an end-stage liver disease [2]. Although many efforts have been placed on a better understanding of the mechanisms implicated in NAFLD pathogenesis, important pathogenic aspects are still not completely understood. Interestingly, recent studies have shown that compositional alterations of conventional gut microbiota, known as dysbiosis, are associated with the development of both obesity and NAFLD [3,4]. Nevertheless, the reasons underlying this causal relationship remain a challenge for our current understanding. Anatomically, the gut and the liver are intimately connected as the majority of the liver blood flow comes directly from the intestinal portal circulation. Thus, intestinal microbiota can exert different effects on liver histology through several direct and indirect mechanisms, including Toll-like receptor (TLR) activation [5]. Upon this recognition, it has been established that TLRs recruit specific adaptor molecules like MyD88 that can initiate downstream signaling events towards the activation of NFκB (Nuclear Factor Kappa-light-chain-enhancer of activated B cells) and MAPK (Mitogen-Activated Protein Kinases), thereby inducing the secretion of inflammatory cytokines, type I interferon (IFN) and different chemokines and antimicrobial peptides [6,7].

Besides the well-documented role of TLR4 and its specific ligand, lipopolysaccharide, in disease pathogenesis in NAFLD [8], other TLRs are emerging as potentially implicated in the development and progression of liver damage in NAFLD. In this sense, research in animal models of obesity-related NAFLD has recently addressed the role of TLR2. The blockade of TLR2 signaling in different in vivo disease models [9,10] has been shown to prevent the development of insulin resistance, intimately associated with NAFLD pathogenesis [11]. Moreover, TLR2 deficient mice on a dietary-induced NASH did not develop steatohepatitis and displayed a lower expression of pro-inflammatory cytokines compared to wild type animals [12], and, furthermore, as recently shown, TLR2 can activate the inflammasome in Kupffer cells, leading to pro-inflammatory cytokine production and NASH development in mice models [13]. These findings are of great interest since TLR2 recognizes a wide range of components of the cell surface of Gram-positive bacteria (lipopeptides, peptidoglycan and lipoteichoic acid), which includes Firmicutes [6]. These have been proposed to be the predominant phyla within the gut microbiota of obese patients on a high fat diet, as well as within NAFLD subjects [3,4,14]. Mechanistically, TLR2 generally forms heterodimers with other TLRs including TLR6, and together can specifically recognize diacylated lipopeptides from Gram-positive bacteria and mycoplasma [15]. Despite the role that TLR2 plays in obesity-related NAFLD, the role of TLR6 in this disease remains unveiled. Therefore, the aim of this study was to comparatively explore the expression and activity (in terms of pro-inflammatory cytokine production) of TLR2 and TLR6 in peripheral blood mononuclear cells (PBMCs) and liver tissue from clinically characterized morbidly obese patients with different degrees of NAFLD severity.

## 2. Results

### 2.1. Baseline Patient Characteristics and Histologic Analysis

Clinical, analytical and anthropometric data was collected from our study group prior to bariatric surgery (summarized in Table 1). Overall, our cohort was mainly composed of female patients (25/40; 62.5%) diagnosed with obesity class III (mean body mass index (BMI) higher than 40 kg/m^2^) and severe obesity-related comorbidities encompassed in the definition of metabolic syndrome [16]. Liver function tests displayed normal mean results in all analyzed parameters including aspartate aminotransferase (AST), alanine aminotransferase (ALT), γ-glutamyl transpeptidase (GGT), alkaline phosphatase (ALP), bilirubin, albumin, platelets and prothrombin time. Liver histology analysis was feasible in 34 of the 40 patients included in the study (Table 2). Clinically, our patients displayed mild liver fibrosis with more specific data depicted as follows: 76.5% had no liver fibrosis or only perisinusoidal or periportal (26/34 F0–F1), 20.6% showed perisinusoidal and portal or periportal fibrosis (7/34 F2) and only one patient displayed bridging fibrosis (2.9% F3). Regarding NAFLD histologic features, our study group was stratified in four groups: (A) 14.7% (5/34) displayed a NAFLD activity index score (NAS) of 0, and, therefore, were considered as normal; (B) 35.3% (12/34) showed an NAS score of 1–2 and were stratified as non-alcoholic fatty liver (NAFL) patients; (C) 41.2% (14/34) had an NAS score between 3 and 4 and therefore were considered as possible or borderline cases of NASH; and (D) 8.8% (3/34) had a NAS score higher than 5, and, therefore, a definite diagnosis of NASH. For the purpose of the study, patients with a possible and a definite diagnosis of NASH were clustered together.

### 2.2. Toll-Like Receptor 2 (TLR2) and TLR6 Expression Profile and Functionality in Peripheral Blood Mononuclear Cells (PBMCs)

**Figure 1:**
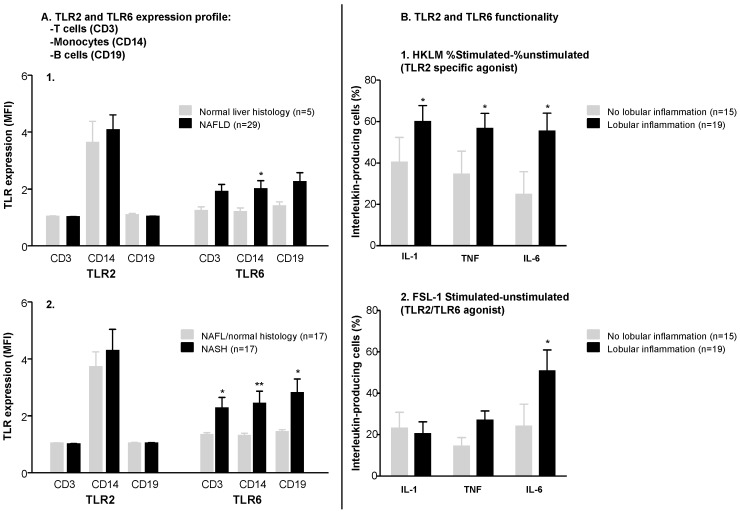
(**A1**) Toll-like receptor 6 (TLR6) expression profile is different in monocytes of non-alcoholic fatty liver disease (NAFLD) patients compared to morbidly obese (MO) patients with a normal liver histology. TLR expression profile in peripheral blood mononuclear cells (PBMCs) of MO patients with and without NAFLD (mean (95% CI)). (TLR6: 1.95 mean fluorescence intensity (MFI) (1.27–2.64) in NAFLD compared to 1.20 MFI (0.84–1.56) in MO with normal histology; *p* < 0.05); (**A2**) TLR6 expression is increased in PBMCs (T cells, monocytes and B cells) of MO patients with a possible or definite diagnosis of non-alcoholic steatohepatitis (NASH) (according to NAFLD activity index). TLR expression profile in PBMCs of MO patients with and without NASH. (TLR6 in T cells: 2.29 MFI (1.44–3.15) in NASH compared to 1.31 MFI (1.68–1.46) in non-NASH; *p* = 0.05. TLR6 in monocytes: 2.45 MFI (1.45–3.45) in NASH compared to 1.28 MFI (1.12–1.45) in non-NASH; *p* = 0.004. TLR6 in B cells: 2.79 MFI (1.75–3.82) in NASH compared to 1.41 MFI (1.26–1.57) in non-NASH; *p* = 0.02). MFI: mean fluorescence intensity; (**B**) intracellular production of pro-inflammatory cytokines is significantly increased in monocytes of morbidly obese patients with lobular inflammation. Patients with lobular inflammation displayed an increased production of IL6 in response to specific stimulation with TLR2 (HKLM) (*p* = 0.01) (**B1**) and TLR6 (FSL-1) (*p* = 0.02) agonists (**B2**). TNFα and IL-1β were also increased in response to stimulation with HKLM (*p* = 0.03 and *p* = 0.01, respectively) (**B1**). * *p* < 0.05. ** *p* < 0.005.

To explore the role of TLR2 and TLR6 in our cohort of clinically characterized patients and healthy controls, we first analyzed their expression, in freshly isolated T-cells, B-cells and monocytes from peripheral blood, using FACS analysis. We found no significant differences in TLR2 and TLR6 expression profile between morbidly obese subjects and healthy voluntaries (data not shown). Nevertheless, interestingly, only TLR6 but not TLR2 was differently expressed in patients with NAFLD compared to morbidly obese subjects with no histological features of liver damage. Thus, the expression of TLR6 in monocytes of patients with NAFLD was significantly higher when compared to those with normal liver biopsy (Figure 1A1). Moreover, in all cells tested, we found a higher expression profile of TLR6 in patients with a possible or definite diagnosis of NASH as compared to those NASH negative (NAFL patients and normal liver histology), (Figure 1A2).

We next decided to study the effects of specific TLR stimulation in freshly isolated peripheral monocytes from patients stratified according to different features of liver histology and in healthy voluntaries. Basal production of pro-inflammatory cytokines from monocytes was increased, as expected, in morbidly obese patients compared to controls (Figure S1). Focusing in morbidly obese patients and according to their liver involvement, we found significant differences in the production of pro-inflammatory cytokines like IL-1β, TNF-α and IL-6 in response to specific TLR2 and TLR2/TLR6 stimulation regarding liver involvement (Figure 1B1,B2). Main differences were found in terms of inflammation. Patients with any grade of lobular inflammation showed an increased IL-6 production in response to specific stimulation with TLR2 and TLR2/TLR6 agonists (HKLM and FSL-1, respectively) (Figure 1B1,B2). These patients also displayed an increased production of TNF-α and IL-1β dependent on TLR2 stimulation (Figure 1B1).

### 2.3. TLR6 and TLR2 Liver Immunohistochemistry and Cytokine Expression Analysis

**Figure 2:**
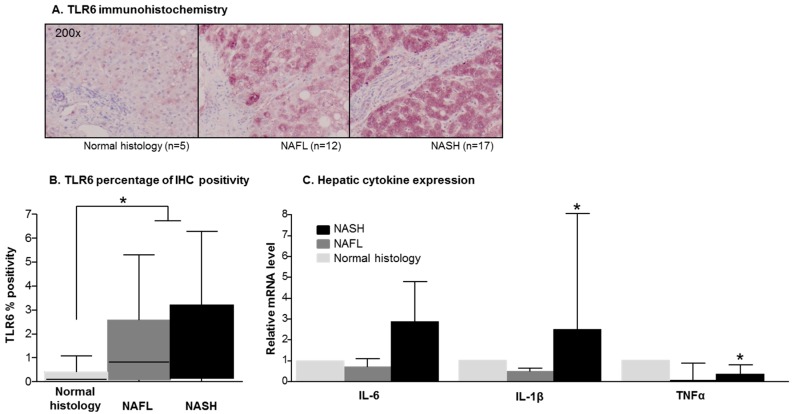
(**A**) Hepatocyte immunohistochemistry mean percentage of expression of TLR6 is enhanced in NAFL and NASH patients compared to morbidly obese with normal histology; (**B**) median percentage of TLR6 immunohistochemistry positivity in liver biopsies. Median positivity is significantly higher in the overall cohort of patients with NAFLD compared to those who displayed normal histology (median (IQR) positivity in NAFLD 0.56 (0.11–2.78) compared to median positivity in normal histology 0.09 (0.01–0.39); *p* = 0.04). No differences were found in the expression of TLR6 in the hepatocytes of NAFL compared to NASH patients; (**C**) hepatic gene expression profile of IL-6, TNFα and IL1β. Relative mRNA levels were analyzed in NAFL and NASH compared to patients with normal liver histology. All three cytokines were more expressed in the livers of patients with NASH compared to patients with normal liver histology (median (IQR): IL-1β: 2.48 (1.04–8.03) in NASH compared to 0.47 (0.38–0.63) in NAFL; *p* = 0.01; TNF: 0.33 (0.15–0.80) in NASH compared to 0.05 (0.02–0.88) in NAFL; *p* = 0.03), although in the case of IL-6, this difference did not reach statistical significance. * *p* < 0.05.

To address the potential role for TLR2 and TLR6 in the liver, we decided to study the expression of both receptors using immunohistochemistry (IHC) in samples corresponding to liver biopsies from our cohort of patients. In this setting and in line with our previous results in peripheral blood cells, TLR6 expression, but not TLR2, was significantly higher in the hepatocytes from the cohort of NAFLD diagnosed patients, compared to those with normal liver histology (Figure 2A). When stratifying patients according to their liver involvement, we found that NASH patients displayed a significantly higher expression of hepatic TLR6 compared to patients with normal liver biopsy, but no differences were found between NAFL and NASH patients (Figure 2B).

Finally, we studied the liver expression of IL-6, TNF-α and IL-1β using RT-qPCR. All three cytokines showed an increased hepatic expression in patients with NASH compared to those with normal liver biopsies or NAFL, with TNF-α and IL-1β, reaching statistical significance (Figure 2C).

## 3. Discussion

Accumulating data shows a preeminent role of TLRs in the pathogenesis of NAFLD. TLRs are pattern recognition receptors that characteristically sense pathogenic microorganisms and bacterial-derived molecules. To date, 10 TLRs have been described in humans that are expressed either in the cell surface (TLR1, TLR2, TLR4, TLR5 and TLR6) or associated to intracellular vesicles (TLR3, TLR7, TLR8 and TLR9). Whereas cell surface TLRs mainly recognize microbial membrane components, intracellular TLRs mostly recognize nucleic acids [6,7]. Only TLR2, TLR4, TLR5 and TLR9 have been associated with the development of NASH, mainly in animal models [8,12,17,18,19,20]. In our study, we found a parallel between liver features of NAFLD and the expression and functionality of TLR6 in PBMCs and hepatocytes. TLR6 expression in B cells, T cells and monocytes was found significantly higher in patients with NASH, compared to those with normal liver biopsies or NAFL, which can reflect the potential of this receptor as a peripheral biomarker of obesity-related NASH. This finding is further supported by an enhanced TLR2/TLR6-dependent cytokine production in peripheral monocytes of patients who display any degree of lobular inflammation. Therefore, we could assume that deregulated TLR6 expression and activity may potentiate the chronic low-grade pro-inflammatory state associated to obesity, in parallel with the events taking place in the liver, which eventually determine NAFLD severity. Nevertheless, we cannot exclude that the deregulated expression and functionality of TLR6 could be a consequence of the pro-inflammatory state related to obesity and the liver disease more than the trigger.

Interestingly, we also found an increased expression of TLR6 concomitant with deregulated pro-inflammatory cytokine expression in the hepatocytes of patients with NAFLD when compared to those morbidly obese patients with normal liver histology. Therefore, we could hypothesize that deregulated TLR6 may not only contribute to the chronic and systemic pro-inflammatory state related to obesity, mirroring the inflammation that is taking place within the liver, but also actively participate in the disease development and progression of obese NAFLD. Moreover, we also found an increased expression of pro-inflammatory cytokines in the liver of patients with NASH, expressing higher levels of TLR6. Despite this being indirect evidence, we believe that this could be explained, at least in part, by an enhanced downstream TLR2/TLR6 activity. Indeed, deregulated TRL6 could potentiate TLR2/TLR6 downstream signaling and promote inflammation independently of TLR2 levels. In this regard, we did not detect changes in TLR2 expression neither in PBMCs nor in hepatocytes, between morbidly obese patients with and without NAFLD or NASH.

Regarding the TLR2/TLR6 signaling axis in our cohort, patients with no definite diagnosis of NASH but borderline had the same peripheral and liver expression of TLR6 and pro-inflammatory cytokines, than those with definite diagnosis of NASH. The lack of inflammation detected by normal circulating IL-6 levels has been suggested as a marker to exclude the diagnosis of NASH [21]. This acquires particular relevance in light of recently published research by Ratziu [22] and Anstee [23] regarding NAFLD progression. Both works agree on the risk of progression towards well-defined fibrosing-NASH among a substantial proportion of patients with NAFL, especially in those without a definite diagnosis of NASH at the beginning of follow up, but with mild lobular inflammation. Thus, we believe that the inflammatory mechanisms dependent on TLR2/TLR6 axis may contribute to the progression towards more severe forms of the disease in morbidly patients with a borderline diagnosis of NASH. Moreover, it is also possible that detecting increased levels of TLR6 expression in peripheral blood (see Figure 1A2) could serve as a non-invasive method to screen for patients that could potentially make progress towards more aggressive forms of obesity-related NAFLD.

## 4. Materials and Methods

### 4.1. Patients

Forty morbidly obese (MO) patients undergoing bariatric surgery were prospectively included in our study from April 2012 to January 2014 as well as 15 healthy controls. Clinical, demographic and anthropometric data were collected and patients were checked-up before inclusion in order to rule out concomitant causes of liver disease. Patients with known history of alcohol consumption (more than 30 g/OH/day in men or 20 g/OH in women), chronic viral hepatitis, inborn errors of metabolism, Wilson’s disease, autoimmune hepatitis, primary biliary cirrhosis or current treatment with steatogenic drugs were excluded. Blood samples were drawn after 12 h overnight fasting the day before bariatric surgery for flow cytometry analysis. Soluble levels of aspartate and alanine aminotransferases (AST and ALT, respectively), γ-glutamyl transpeptidase (GGT), alkaline phosphatase (AP), albumin, platelets, total cholesterol, HDL-cholesterol, triglycerides, 25-OH-vitamin D, glucose, insulin and glycosylated hemoglobin were determined. Insulin resistance was evaluated using the homeostasis model assessment (HOMA) [24].

Written informed consent was obtained from all patients and the study was conducted conformed to the ethical guidelines of the Helsinki Declaration and with approval of the local Ethics Committee.

### 4.2. TLR Expression Profile in PBMCs

Cell surface expression of TLR2 and TLR6 was assessed on peripheral blood T cells, B cells and monocytes by flow cytometry. Cells collected into sodium heparin tubes were incubated with fluorochrome-conjugated anti-human CD19, anti-human CD3 and anti-human CD14 (BD Biosciences, San Diego, CA, USA) to identify B cells, T cells and monocyte populations, respectively, and with fluorochrome-conjugated anti-human TLR2 and TLR6 or mouse IgG2a isotype control (eBioscience, San Diego, CA, USA) for 20 min in the dark. After being washed, the cells were re-suspended in 1% paraformaldehyde. Expression of TLRs was gated and analyzed by flow cytometry (FACSDiva Software; BD Biosciences) as mean fluorescence intensity (MFI) with regard to isotype control signal.

### 4.3. Assessment of TLR Function in Circulating Monocytes

Whole blood cells collected in sodium heparin tubes were polyclonally stimulated for 18 h with specific agonists for human TLR2 (HKLM) and TLR2/TLR6 heterodimer (FSL-1) (InvivoGen, San Diego, CA, USA) in the presence or absence of brefeldin A (Sigma-Aldrich, St. Louis, MO, USA) in polypropylene tubes, as previously described [25]. After culture, cells were stained with FITC-conjugated anti-human CD14 (BD Biosciences) to identify the monocyte population. Then, red blood cells were lysed with FACS lysing solution (BD Biosciences) for 10 min. After being washed, the cells were permeabilised and intracellularly stained with phycoerythrin-labelled monoclonal antibodies against cytokines: interleukin (IL)-1β, tumor necrosis factor (TNF)-α, IL-6; and analyzed by flow cytometry using FACSDiva Software.

### 4.4. Histology Assessment

Liver biopsies were obtained during surgery. Despite the unavoidable inter- and intra-observer variability of the results of liver histology, this technique continues to be the gold standard in the diagnosis of NAFLD. Biopsy slides stained in haematoxylin and eosin (H&E) and Masson trichrome were reviewed by an expert pathologist. Four histological features (steatosis, lobular inflammation, hepatocellular ballooning and fibrosis) were evaluated according to Brunt’s classification [26]. Subsequently, liver biopsies were categorized following the scoring system proposed by Kleiner et al. [27].

### 4.5. Immunohistochemistry

Protein expression of TLR2 and TLR6 was determined in paraffin-embedded liver sections using specific antibodies at a concentration of 1:100 (LifeSpan BioSciences Inc., Seattle, WA, USA). First, samples were baked for 30 min, dewaxed in xylene, and rehydrated in a graded series of ethanol solutions. All procedures were done according to standard protocols with EnVision+ System HRP (Dako, Glostrup, Denmark). Finally, samples were incubated with Vector Vip substrate (Vector Laboratories, Inc., Burlingame, CA, USA) for purple color development. Images were taken with a 10× objective from an AXIO Imager A1 microscope (Carl Zeiss AG, Jena, Germany). Quantification of staining intensity of area for each sample was calculated using FRIDA software, a custom open source image analysis software package (FRamework for Image Dataset Analysis, available at: http://sourceforge.net/projects/fridajhu/).

### 4.6. Cytokine Gene Expression Analysis

Total RNA was isolated from liver tissue using TRIzol reagent (Invitrogen, Carlsbad, CA, USA). Two µg of total RNA were treated with DNAse (Invitrogen) and reverse transcribed into cDNA using M-MLV Reverse Transcriptase (Invitrogen). Then, qPCR was performed using iQ™ SYBR^®^ Green Supermix (BioRad, Hercules, CA, USA) using the CFX Connect™ RT-PCR Detection System (BioRad) to determine the expression of IL-6, IL-1β and TNF-α. Expression levels were normalized to that of GAPDH mRNA in each sample.

### 4.7. Statistical Analysis

Normally distributed values were analyzed by Student’s *t*-test, while the Mann–Whitney test was used for non-parametric values. Categorical comparisons were performed using Χ^2^ or Fisher’s exact test, as appropriate. Statistical significance was considered when *p* < 0.05.

## 5. Conclusions

In summary, we found deregulated expression of TLR6 with a concomitantly activated downstream TLR2/TLR6 activity towards the expression of pro-inflammatory cytokines in peripheral blood cells and in hepatocytes from obesity-related NAFLD patients. These data suggest a role for TLR2/TLR6 dependent signaling in the pathogenesis of NASH with deregulated TLR6 potentiating TLR2/TLR6 downstream activity in morbidly obese patients, although this finding needs to be further confirmed on a larger sample size study. Finally, TLR6 overexpression in peripheral blood cells can offer the opportunity to develop non-invasive tools to support the diagnosis of obesity-related NASH.

## Figures and Tables

**Table 1:** Clinical, anthropometrical and analytical data of the study population. Comparison of patients’ characteristics according to the presence of non-alcoholic steatohepatitis in liver biopsy. Patients are clustered according to histology: non-alcoholic steatohepatitis (NASH) group includes possible and definite cases of steatohepatitis; NAFL group includes patients in which steatohepatitis has been ruled out. Qualitative data is presented as total number and percentage in brackets. Quantitative data is presented as mean and standard deviation in brackets.

Patients’ Characteristics	Overall (*n* = 40)	Non-NASH (*n* = 17)	NASH (*n* = 17)	*p*
Male/female	15/25 (37.5/62.5)	3/14 (17.6/82.4)	11/6 (64.7/35.3)	0.007
Age	43.78 (10.33)	41.00 (9.01)	42.76 (10.9)	NS
Metabolic syndrome	23 (57.5)	6 (35.3)	13 (76.5)	0.02
Obstructive sleep apnea	27 (67.5)	8 (47.1)	15 (88.2)	0.01
BMI (kg/m^2^)	48.17 (4.93)	49.52 (5.7)	47.79 (3.2)	NS
Waist circumference (cm)	139.48 (11.24)	135.94 (9.5)	143.47 (12.4)	NS
Hip circumference (cm)	144.61 (10.41)	146.47 (11.9)	143.62 (8.1)	NS
Systolic blood pressure (mm Hg)	136.27 (13.99)	132.81 (9.2)	142.56 (16.4)	0.05
Diastolic blood pressure (mm Hg)	82.89 (14.44)	83.25 (15.2)	82.94 (16.2)	NS
LDL-Cholesterol (mg/dL)	102.38 (33.11)	109.13 (31.9)	91.44 (31.7)	NS
HDL-Cholesterol (mg/dL)	40.35 (10.14)	43.80 (10.5)	37.31 (8.8)	NS
Total cholesterol (mg/dL)	179.97 (40.38)	184.24 (39.4)	172.63 (41.2)	NS
Tryglicerides (mg/dL)	167.57 (93.19)	128.44 (62.9)	208.30 (107.5)	0.02
Homocysteine (µmol/L)	11.29 (3.49)	11.72 (4.6)	10.84 (2.64)	NS
cCRP (mg/L)	9.82 (10.73)	13.34 (15.1)	8.08 (5.4)	NS
AST (U/L)	28.11 (12.40)	23.00 (7.6)	34.88 (14.5)	0.007
ALT (U/L)	33.51 (18.52)	23.53 (7.5)	45.59 (21.8)	0.001
GGT (U/L)	32.08 (21.12)	29.50 (16.4)	35.41 (26.3)	NS
Alkaline phosphatase (U/L)	70.89 (17.86)	72.69 (18.6)	68.65 (18.8)	NS
Bilirubin (mg/dL)	0.77 (0.31)	0.67 (0.2)	0.85 (0.4)	NS
Albumin (mg/dL)	4.16 (0.27)	4.18 (0.2)	4.15 (0.3)	NS
Ferritin (mg/dL)	159.60 (160.38)	99.88 (79.3)	261.36 (201.5)	0.02
HbA1c (%)	6.22 (1.58)	5.48 (0.3)	6.99 (2.1)	0.008
Platelets (10^3^/µL)	240.87 (62.04)	269.35 (58.9)	213.53 (51.4)	0.006
Prothrombin time (%)	77.79 (9.08)	75.71 (7.8)	78.82 (7.1)	NS
HOMA index	4.01 (4.24)	3.29 (1.5)	5.76 (5.7)	NS
25-OH-Vit D (ng/mL)	15.95 (5.74)	15.18 (6.0)	15.47 (4.0)	NS

NS: non-significant; BMI: body mass index; LDL: low-density lipoprotein; HDL: high-density lipoprotein; CRP: C reactive protein; AST: aspartate aminotransferase; ALT: alanine aminotransferase; GGT: γ-glutamyl transpeptidase; HOMA: homeostasis model assessment; NS: non-significant.

**Table 2:** Liver histology classification. Liver biopsies were evaluated following non-alcoholic fatty liver disease (NAFLD) features proposed by Brunt and subsequently classified according to the NAFLD activity index validated by Kleiner. Fibrosis was evaluated in biopsy slides stained in Masson trichrome. Data are presented as n and percentage in parenthesis.

Liver Histology according to Brunt’s Classification	
Steatosis grade (%)	
0: minimal or <5%	10 (25)
1: 5%–33%	14 (35)
2: >33%–66%	3 (7.5)
3: >66%	7 (17.5)
Lobular inflammation grade (%)	
0: no foci	15 (44.1)
1: <2 foci per 200 × field	18 (52.9)
2: 2–4 foci per 200 × field	0 (0)
3: >4 foci per 200 × field	1 (2.9)
Ballooning grade (%)	
0: none	23 (67.6)
1: few balloon cells	4 (11.8)
2: many cells, prominent ballooning	7 (20.6)
NAFLD activity index score (NAS) (%)	
0: normal liver histology	5 (14.7)
1–2: not steatohepatitis	12 (35.3)
3–4: possible or borderline steatohepatitis	14 (41.2)
5–8: definite steatohepatitis	3 (8.8)
Fibrosis grade (%)	
F0: none	5 (14.7)
F1: perisinusoidal or periportal	21 (61.8)
F2: perisinusoidal and portal/periportal	7 (20.6)
F3: bridging fibrosis	1 (2.9)
F4: cirrhosis	0 (0)

## Supplementary Materials

Supplementary materials can be found at www.mdpi.com/1422-0067/17/11/1878/s1.

## Abbreviations

NAFLD	Non-alcoholic fatty liver disease
NASH	Non-alcoholic steatohepatitis
TLR	Toll-like receptor
PBMCs	Peripheral blood mononuclear cells
MO	Morbidly obese
AST	aspartate aminotransferase
ALT	alanine aminotransferase
GGT	γ-glutamyl transpeptidase
AP	alkaline phosphatase
HOMA	homeostasis model assessment
MFI	Mean fluorescence intensity
IL	Interleukin
TNF	Tumor necrosis factor
NAFL	Non-alcoholic fatty liver
IHC	Immunohistochemistry
