# Supplementary material for: Increased Expression Profile and Functionality of TLR6 in Peripheral Blood Mononuclear Cells and Hepatocytes of Morbidly Obese Patients with Non-Alcoholic Fatty Liver Disease
Source: Int J Mol Sci. 2016 Nov 10;17(11):1878. doi: 10.3390/ijms17111878 (PMC5133878; doi:10.3390/ijms17111878)
Supplement: Supplementary file 1 [file ijms-17-01878-s001.pdf]

Supplementary Materials: Increased Expression Profile and Functionality of TLR6 in Peripheral Blood Mononuclear Cells and Hepatocytes of Morbidly Obese Patients with Non-Alcoholic Fatty Liver Disease

María Teresa Arias-Loste, Paula Iruzubieta, Ángela Puente, David Ramos, Carolina Santa Cruz, Ángel Estébanez, Susana Llerena, Carmen Alonso-Martín, David San Segundo, Lorena Álvarez, Antonio López Useros, Emilio Fábrega, Marcos López-Hoyos and Javier Crespo

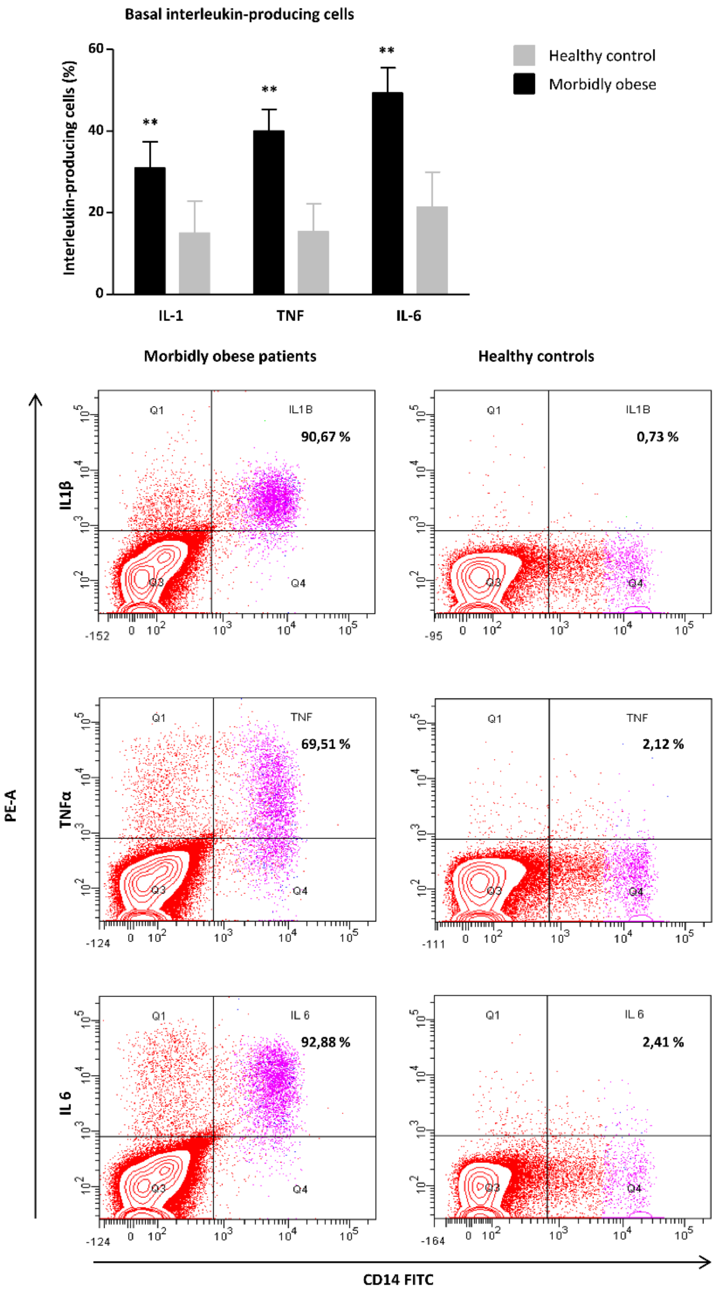

Figure S1. Basal intracellular production of pro-inflammatory cytokines. ** $p < 0.005$.
